# Flourishing mental health despite disabling chronic pain: Findings from a nationally representative sample of Canadians with arthritis

**DOI:** 10.1371/journal.pone.0291722

**Published:** 2023-10-11

**Authors:** Esme Fuller-Thomson, Denise J. Marshall, Matthew Moses, Sally Abudiab

**Affiliations:** 1 Factor-Inwentash Faculty of Social Work, University of Toronto, Toronto, Canada; 2 Institute for Life Course & Aging, University of Toronto, Toronto, Canada; 3 Department of Family and Community Medicine, University of Toronto, Toronto, Canada; Singapore General Hospital, SINGAPORE

## Abstract

This study aims to determine the prevalence of, and factors associated with, the “absence of psychiatric disorders” (APD) and “complete mental health” (CMH) among individuals with arthritis who report disabling chronic pain. There are three aspects of CMH: a) APD; b) happiness and/or life satisfaction in the past month on a daily or almost daily basis, and c) high levels of psychological and social well-being. A secondary analysis of a nationally representative sample (n = 620) of individuals with arthritis who report chronic and debilitating pain was conducted. Data were drawn from the Canadian Community Health Survey-Mental Health. The results of this study indicate that many people with arthritis who are living with disabling chronic pain are free of psychiatric disorders (76%) and are in CMH (56%). Factors associated with higher odds of APD and CMH among the sample include having a confidant, being free from insomnia, and having no lifetime history of major depressive disorder and/or generalized anxiety disorder. White respondents were almost 3-fold more likely to be in a state of CMH compared to racialized individuals. Respondents in the top 50% of household incomes were almost 4-fold more likely to be APD compared to the lowest 10%. In conclusion, many individuals with arthritis have excellent mental health despite disabling pain. Clinicians should be attuned to the mental health of their patients, with particular focus on those who may be more vulnerable to adverse mental health outcomes, such as racialized individuals, those in impoverished households, and those who lack social support.

## Introduction

Mental health problems, such as mood, anxiety disorders, suicidality, and substance use disorders have been linked to arthritis in many previous studies [1–4]. The prevalence of major depression among those with arthritis ranges from approximately 10% in representative community samples [1,5] to 40% in clinical samples of those with rheumatoid arthritis [6,7]. Psychological well-being is lower in those with arthritis than those without, particularly in the context of disabling pain [8].

In the general population, chronic pain and functional limitations are both associated with poorer mental health and well-being [9–11]. In the context of arthritis, pain is often not sufficiently managed [12,13]. Chronic pain related to arthritis can undermine mental health. Among those with arthritis, chronic pain that limits most activities doubles the odds of depression in comparison to those whose pain does not limit activities [5]. Individuals with arthritis who have chronic pain experience slower remission from depression in comparison to those with arthritis whose pain is not chronic [14].

Research on mental health among individuals with arthritis has largely focused on mental illness [15,16]. In a paradigm shift, the current research moves away from a deficit-focused approach to mental health among individuals with arthritis, and instead uses a strengths-based perspective to explore factors associated with resilience in individuals with arthritis who are experiencing chronic and disabling pain. Resilience is defined as “a positive adaptation after stressful situations and it represents mechanisms of coping and rising above difficult experiences i.e…resists the negative impact of stressors” (P. S.226) In the current study, the authors suggest that individuals who manage to retain excellent mental health despite chronic and disabling pain are demonstrating resilience. For the operationalization of excellent mental health, we draw upon Keyes’ measure of mental flourishing, known as “complete mental health” (CMH) [17]. CMH encapsulates a more holistic view of mental health, combining the absence of psychiatric disorder with the presence of mental well-being. The model of CMH utilized in the current study is comprised of three components: 1) the absence of psychiatric disorders (e.g., depression, anxiety, bipolar disorder, suicidality, and drug or alcohol dependence); 2) the presence of emotional well-being and high levels of psycho-social functioning; and 3) high levels of happiness and/or life satisfaction.

The current research has two objectives: 1) to determine the prevalence of “absence of psychiatric disorders” (APD) and CMH among individuals with arthritis who are experiencing disabling chronic pain; and 2) to identify factors associated with APD and CMH among those with arthritis who are in debilitating pain.

We are particularly interested in whether these positive mental health outcomes are less common among racialized individuals and those living in poverty. There is a large literature on physical health inequities among those with arthritis indicating that racialized individuals with arthritis have greater pain severity and higher levels of disability than their white peers [18], and those with lower socioeconomic status have worse outcomes and greater disabilities than the more affluent [19]. Furthermore, many studies have highlighted inequities in mental health among racialized and low-income individuals, largely due to less access to mental health services and poorer quality of mental health care [20,21]. Our research will help establish if mental health inequities are also apparent when the sample is restricted to those arthritis patients with disabling pain.

## Methods

The current study was undertaken using nationally representative data from the 2012 Canadian Community Health Survey—Mental Health (CCHS-MH), and has been described fully elsewhere [22,23]. The target population of the CCHS-MH includes 97% of the population who are over the age of 15 and reside in the 10 Canadian provinces. The response rate was 68.9% nationally [24]. The analysis reported here was a secondary data analysis of a public use data set which had been stripped of any individual identifiable information and therefore the University of Toronto Ethics Board did not require the authors to submit this study through the research ethics review process. The original ethics procedure was conducted by Statistics Canada, the Canadian equivalent of the US Census Bureau. Informed consent was obtained by Statistics Canada either by telephone or in-person depending on the mode of data gathering (approximately 86% of the interviews were in-person).

### Sample

A sub-sample of the full CCHS-MS dataset was restricted to those with arthritis who reported debilitating chronic pain. Persons under age 18 and those with missing data on arthritis, chronic pain or any of the other variables used in the fully adjusted model were excluded from the analysis. The final sample size was 620 respondents.

### Measures

#### Arthritis

Participants were asked if they had been diagnosed with arthritis by a health care professional.

#### Debilitating chronic pain

Participants were asked two questions: 1) “Are you usually free of pain or discomfort” (no/yes); and 2) “How many activities does your pain or discomfort prevent?” (none, a few, some, most). Those who reported no to question 1 and ‘most’ to question 2 were defined as having debilitating chronic pain.

#### Outcome variable

Two different outcome measures were evaluated; (a) APD in the past year was based on no suicidal ideation in the past year nor depressive episode, anxiety disorders, bipolar disorders, nor alcohol or drug dependence including cannabis and other drugs. World Health Organization’s version of the Composite International Advances in Preventive Medicine Diagnostic Interview (WHO-CIDI), a structured diagnostic interview that generates past-year diagnosis according to the Diagnostic and Statistical Manual of Mental Disorders, Fourth Edition (DSM-IV) and the international Classification of Disease (ICD-10) was used to derive these variables. Finally, (b) CMH, measured as a binary variable, consisted of three parts: (1) the absence of psychiatric disorders in the past year, as was described above; (2) emotional wellbeing (i.e., life satisfaction and/or happiness), and (3) social and psychological well-being. The latter two were assessed using the Mental Health Continuum—Short Form (MHC-SF) [24]. The MHC-SF examines positive mental health using 14-items that assesses psychological well-being (e.g., during the past month, how often did you feel that you liked most parts of your personality?), emotional well-being (e.g., during the past month, how often did you feel: happy and/or satisfied with your own life?), and social well-being (e.g., during the past month, how often did you feel that you had something important to contribute to society?) [24,25]. The MHC-SF has well established psychometric properties [25]. Respondents were categorized as being in CMH if they stated at least 1 of the 2 measures of emotional well-being (i.e., happiness and/or life satisfaction in past year) and a minimum of 6 of the 11 measures of psychological and/or social wellbeing “every day” or “almost every day” during the past month in tandem with the absence of any of the above listed forms of mental illness in the past year. Additional information can be found at Statistics Canada [24]. Our version was slightly modified by removal of one-question from the original 14-item instrument. The original version had included the question “interested in life?” in addition to “happy” and “satisfied with life” in the “emotional well-being” category. We felt that it was possible to be interested in life without being in optimal mental health and therefore removed it from the measure which resulted in the instrument having only 13 items now. Furthermore, in a previous study of the psychometric properties of the MHC-SF, ‘‘interest in life” had markedly lower factor loading on emotional well-being than either ‘‘happy” or ‘‘satisfaction with life” [25]. The internal consistency (Cronbach’s alpha) for the 13-items was high (0.89).

#### Socio-demographic variables

Participants were asked about their sex (male versus female), self-reported race (Non-Aboriginal white versus racialized and/or Aboriginal), age (in decades), and socioeconomic position (SEP). SEP was calculated based on the highest level of education completed (less than high school, high school, some post-secondary school, post secondary). In line with Statistics Canada’s measure of household income, this study used a ratio associated with the national low-income cut-off, which takes into account the quantity of people who reside in a home and the size of the neighbourhood (lowest 10% 11–50%; top 50% of household income). Due to confidentiality issues, the CCHS-MH does not release information on specific race/ethnicities.

#### Social support

Participants were asked about their marital status and having an advisor/confidant available for critical life decisions and situations. Marital status was measured according to the following two categories: Married/common-law versus divorced/widowed/never married. Having a confidant available for critical life decisions and experiences was ascertained using those who agreed or strongly agreed to the statement “There is someone I could talk to about important decisions in my life.”

#### Adverse childhood experiences

Two adverse childhood experiences were investigated. *Childhood Sexual Abuse (CSA)* was measured by the questions “How many times did an adult force you or attempt to force you into any unwanted sexual activity, by threatening you, holding you down or hurting you in some way?" (never vs ever). *Childhood Physical Abuse (CPA)* was assessed by asking respondents if an “adult had slapped them on the face, head or ears, or hit or spanked them with something hard to hurt them at least three times and/or pushed, grabbed, shoved or threw something at them to hurt them at least three times and/or an adult had at least once kicked, bit, punch, choked, burned, or physically attacked them.”

#### Lifetime mental health factors

Participants were asked about lifetime mental health diagnoses include anxiety and depression. Individuals were categorized as having an anxiety disorder if they met the WHO-CIDI criteria for Generalized Anxiety Disorder at some point in their lives. Respondents who met the criteria for lifetime Generalized Anxiety Disorder reported “1) excessive anxiety and worry and anxiety about at least two different events or activities that lasted at least six months; 2) finding it difficult to control the worry; 3) the anxiety and the worry were associated with three or more of the symptoms associated with anxiety; 4) the focus of the anxiety and worry was not confined to features of an Axis 1 disorder; and 5) the anxiety, worry, or physical symptoms caused clinically significant distress or significant impairment in social, occupational, or other important areas of functioning” [24].

Respondents were categorized as having a depressive disorder if they met the WHO-CIDI lifetime criteria for Major Depressive Episode. “Respondents who meet the criteria reported: 1) two weeks or longer of depressed mood or loss of interest or pleasure and at least five symptoms associated with depression which represent a change in functioning; 2) that symptoms cause clinically significant distress or impairment in social, occupational or other important areas of functioning; and 3) that symptoms are not better accounted for by bereavement or symptoms last more than two months or the symptoms are characterised by a marked functional impairment, preoccupation with worthlessness, suicidal ideation, or psychomotor retardation.” [24]. The WHO-CIDI has strong psychometric properties and excellent validity and reliability [26].

#### Lifetime substance dependence

Participants were asked about lifetime alcohol and/or drug dependence based upon WHO-CIDI scales. To be defined as substance dependent the respondent must report a minimum of three of the following symptoms: tolerance, withdrawal, increased consumption, attempts to quit, time lost, reduced activities, continued drinking and “2) a maladaptive pattern of alcohol use as manifested by three (or more) symptoms occurring at any time in the same 12-month period” [24].

### Statistical analyses

Frequencies were provided for both outcome variables (i.e., APD and CMH) and all the characteristics described above. Two sets of bivariate analyses were conducted using chi-square tests for categorical variable (e.g., sex, ethnicity) and independent t-tests for continuous variables (e.g., age) The first set of crosstabs examined each variable by APD status, and the second set of bivariate analyses compared each characteristic by CMH status. Two logistic regression analyses were conducted, one with APD as the outcome and one with CMH as the outcome. All the variables described above were included in each logistic regression analysis.

Because the CCHS-MH uses a multi-stage sampling design, sample weights that were created by Statistics Canada were needed to take into account the unequal probability of selection of respondents in different provinces and the potential bias due to nonresponse. All prevalences and odds ratios are based on weighted data in order for the sample to be representative of community dwelling adults in Canada. This weighting variable was then rescaled to a mean of 1 for this sample, which is the standardized technique of normalizing weights so as to avoid falsely narrowing the confidence interval. Sample sizes are presented in their unweighted form.

## Results

Table 1 presents the profile and prevalence of respondents with no mental illness in the past year and prevalence of complete mental health in the past month by sociodemographic and life history characteristics in a sample of Canadians with arthritis who reported chronic and debilitating pain (n = 620). Three-quarters (76%) of those with arthritis and disabling chronic pain were APD and more than half (55.6%) were in CMH.

**Table 1:** Profile and prevalence of respondents with no psychiatric disorders in the past year and the prevalence of complete mental health in the past month by sociodemographic and life history characteristics in a sample of Canadians with arthritis who reported chronic and debilitating pain (n = 620).

	Unweighted N	Weighted frequencies of total sample (%)	Weighted % of respondents with no psychiatric disorder	Weighted % of respondents with complete mental health
**OUTCOMES OF INTEREST**				
**Absence of psychiatric disorder**				
Mentally ill/suicidal	140	24.0%		
Not mentally ill/suicidal	480	76.0%		
**Complete mental health status**				
Not complete MH	294	44.4%		
Complete MH	326	55.6%		
**SOCIODEMOGRAPHIC CHARACTERISTICS**				
**Sex**				
Male	238	35.1%	78.8%	55.8%
Female	382	64.9%	74.4%	55.5%
**Ethnicity**				
Racialized	80	14.2%	51.1%***	27.3%***
White	540	85.8%	80.1%	60.3%
**Education**				
No post-secondary degree	328	48.9%	80.5%**	59.2%+
Has post-secondary degree	292	51.1%	71.60%	52.10%
**Household Income**				
Lowest 10% of households	138	21.7%	59.3%***	39.6%***
11–50%	281	41.4%	75.8%	52.7%
Top 50% of household’s income	201	36.9%	86.0%	68.4%
**CHILDHOOD ADVERSITIES**				
**Physical abuse during childhood**				
No	393	63.1%	84.9%***	63.0%***
Yes	227	36.9%	60.3%	43.0%
**Sexual abuse during childhood**				
Never attempted forced sex, possible touching	507	84.3%	81.8%***	60.0%***
Attempted forced sex	113	15.7%	44.3%	31.6%
**SOCIAL SUPPORT**				
**Marital status**				
Single/divorced/widow	343	43.0%	71.1%*	46.8%***
Married/common-law	277	57.0%	79.7%	62.1%
**Presence of a confidant**				
No	48	9.7%	23.3%***	8.3%***
Yes	572	90.3%	81.6%	60.7%
**COPING STRATEGIES**				
**Sleep problems**				
Never to some sleep problems	313	54.6%	89.7%***	70.4%***
Most or all the time sleep problems	307	45.4%	59.4%	37.7%
**Extent of religious or spiritual values providing strength**				
Not at all	110	18.5%	73.9%	48.2%+
Yes, it does	510	81.5%	76.4%	57.2%
**LIFETIME HISTORY OF MENTAL ILLNESS/ SUBSTANCE ABUSE**				
**Drug and alcohol abuse during lifetime**				
Neither drug nor alcohol abuse	433	75.2%	77.0%	57.5%
Either/both drug and alcohol abuse—lifetime	187	24.8%	72.7%	50.0%
**Ever lifetime major depressive disorder**				
Never in life	473	76.9%	90.8%***	67.9%***
Yes—lifetime	147	23.1%	26.6%	14.7%
**Ever lifetime generalized anxiety disorder**				
Never in life	483	77.0%	89.7%***	65.2%***
Yes—lifetime	137	23.0%	30.1%	23.8%

*** indicates p < .001;

** indicates p < .01;

* indicates p < .05;

^+^ indicates p < .10.

The mean age of those in APD was 62.4 (SD = 12.8), which was significantly higher than those who were not in APD (54.7; SD = 12.8; p < .001). The mean age of those in CMH (61.9; SD = 12.8) was significantly higher than those who were not in CMH (58.9; SD = 13.7; p = 0.005) As shown in Table 1, the prevalence of both APD and CMH was significantly higher for married individuals (79.7% vs 71.1%, p < 0.05, and 62.1% vs 46.8%, p < 0.001, respectively), white respondents compared to racialized respondents (80.1% vs 51.1%, p < 0.001, and 60.3% vs 27.3%, p < 0.001), those with higher income (86.0% vs 59.3%, p < 0.001, and 68.4% vs 39.6%, p < 0.001) and those with no history of childhood physical (84.9% vs 60.3%, p < 0.001, and 43.0% vs 63.0%, p < 0.001) or sexual abuse (81.5% vs 44.3%, p < 0.001, and 60.0% vs 31.6%, p < 0.001). The prevalence of both APD and CMH was also higher among those who self-reported never to sometimes having sleep problems compared to those who self-reported most of the time having sleep problems (89.7% vs 59.4%, p < 0.001, and 70.4% vs 37.7%, p < 0.001) and having a confidant compared to not having a confidant (81.6% vs 23.3%, p < 0.001, and 60.3% vs 8.3%, p < 0.001). Those without a post-secondary degree were more likely to be APD than those with a post-secondary degree (80.5% vs 71. 60%; p < 0.01). A similar trend for education was evident with respect with CMH but it failed to reach statistical significance, p < .10).

Table 2 provides the results of two logistic regression models examining the odds and 95% confidence intervals (CI) of 1) being free of psychiatric disorders in the past year and 2) achieving complete mental health in the past month in a sample of Canadians with arthritis who reported chronic and debilitating pain. The odds of APD were more than 3-fold higher in individuals in the top 50% of household incomes (OR = 3.67, CI = 1.55, 8.68), and more than double among those whose income was between 10% and 49% (OR = 2.84, CI = 1.27, 6.36), compared to those in the lowest 10% of household incomes.

**Table 2:** Odds and 95% confidence intervals (CI) of 1) being free of psychiatric disorders in the past year and 2) achieving complete mental health in the past month in a sample of Canadians with arthritis who reported chronic and debilitating pain (n = 620).

	Odds ratio with 95% CI—absence of psychiatric disorder	Odds ratio with 95% CI for complete mental health
**SOCIODEMOGRAPHIC CHARACTERISTICS**		
**Household Income**		
Lowest 10% of households (ref.)	1.00 (Ref)	1.00 (Ref)
11–50%	2.84 (1.27, 6.36)	1.21 (0.71, 2.08)
Top 50% of household’s income	3.67 (1.55, 8.68)	1.77 (1.002, 3.13)
**Sex**		
Male (ref.)	1.00 (Ref)	1.00 (Ref)
Female	1.98 (0.98, 4.01)	1.51 (0.95, 2.39)
**Ethnicity**		
Racialized (ref.)	1.00 (Ref)	1.00 (Ref)
White	1.28 (0.52, 3.12)	2.83 (1.48, 5.41)
**Age (per decade)**	0.89 (0.69, 1.16)	0.78 (0.66, 0.92)
**Education**		
No post-secondary degree (ref.)	1.00 (Ref)	1.00 (Ref)
Has post-secondary degree	0.79 (0.42, 1.50)	0.80 (0.53, 1.21)
**SOCIAL SUPPORT**		
**Marital Status**		
Single/divorced/widowed	1.00 (Ref)	1.00 (Ref)
Married/common-law	1.02 (0.54, 1.94)	1.35 (0.88, 2.06)
**Presence of a confidant**		
No (ref.)	1.00 (Ref)	1.00 (Ref)
Yes	5.81 (2.36, 14.26)	6.10 (2.10, 17.74)
**CHILDHOOD ADVERSITIES**		
**Physical abuse during childhood**		
Yes (ref.)	1.00 (Ref)	1.00 (Ref)
No	1.60 (0.82, 3.15)	0.98 (0.62, 1.55)
**Sexually abused during childhood**		
Attempted forced sex (ref.)	1.00 (Ref)	1.00 (Ref)
Never attempted forced sex, possible touching	1.75 (0.73, 4.17)	1.22 (0.63, 2.38)
**COPING STRATEGIES**		
**Sleep problems**		
Most or all the time sleep problems (ref.)	1.00 (Ref)	1.00 (Ref)
Never to some sleep problems	3.31 (1.76, 6.21)	2.32 (1.55, 3.47)
**Extent of religious or spiritual values providing strength**		
Not at all (ref.)	1.00 (Ref)	1.00 (Ref)
Yes, it does	0.73 (0.32, 1.63)	1.40 (0.84, 2.35)
**LIFETIME HISTORY OF MENTAL ILLNESS/ SUBSTANCE ABUSE**		
**Ever lifetime major depressive disorder**		
Yes (ref.)	1.00 (Ref)	1.00 (Ref)
No	18.43 (9.10, 37.31)	8.15 (4.44, 14.94)
**Ever lifetime generalized anxiety disorder**		
Yes (ref.)	1.00 (Ref)	1.00 (Ref)
No	16.64 (8.40, 32.97)	2.70 (1.60, 4.55)
**Drug and alcohol abuse during lifetime**		
Either/both drug and alcohol abuse—lifetime (ref.)	1.00 (Ref)	1.00 (Ref)
Neither drug nor alcohol abuse	0.55 (0.26, 1.17)	1.12 (0.67, 1.87)

Those in the highest 50% of household incomes were 77% more likely to be in a state of CMH compared to those in the lowest 10% of household incomes. White respondents were more than twice as likely to be in CMH (OR = 2.83, CI = 1.48, 5.41) compared to racialized respondents. Individuals with a confidant were more than 5-fold more likely to be APD (OR = 5.81, CI = 2.36, 14.26) and in CMH (OR = 6.10, CI = 2.10, 17.74) than those without at least one confidant. Individuals without sleep problems were more than twice as likely to be in APD (OR = 3.31, CI = 1.76, 6.21) and in CMH (OR = 2.32, CI = 1.55, 3.47). Individuals who never previously had an episode of major depressive disorder were much more likely to be APD (OR = 18.43, CI = 9.10, 37.31) and in CMH (OR = 8.12, CI = 4.44, 14.94) than those who had ever had depression. Similarly, individuals without a lifetime episode of generalized anxiety were more than 16-fold more likely to be APD (OR = 16.64, CI = 8.40, 32.97) and more than twice as likely to be in CMH (OR = 2.70, CI = 1.60, 4.55) compared to those without a history of anxiety.

## Discussion

The findings of this study indicate a high level of resilience among a nationally representative sample of Canadians with arthritis who reported that most of their activities were restricted due to pain. Fully three quarters of the sample were free of psychiatric disorders in the past year, including depression, generalized anxiety disorders, bipolar disorders, serious suicidal thoughts, and substance dependence, despite their debilitating pain. More than half (56%) went beyond just being free of psychiatric disorders to achieving complete mental health, which is indicative of happiness and/or life satisfaction in addition to psychological and social well-being. These findings bring a hopeful message to those living with disabling pain and arthritis and their families as well as to clinicians addressing their physical and mental health care needs.

The resiliency observed among in the current study may not be unique to those with arthritis. In another nationally representative Canadian survey of community dwelling adults, 57% of individuals with chronic and disabling pain due to any health condition were in complete mental health [27]. This suggests that those with arthritis and chronic and disabling pain have comparable levels of CMH to other Canadians who have chronic and disabling pain due to other health conditions.

On a less positive note, our findings indicate that those living in the poorest households and racialized respondents have a lower prevalence of APD or CMH than their white and more affluent peers. The magnitude of these health inequities is substantial. The observed negative association between poverty and both APD and CMH exists despite the universal healthcare system in Canada that is available to support patients’ arthritis-related needs, as well as their mental health problems. This suggests that socioeconomic disadvantage may be playing a role through other avenues than solely health care access. For example, individuals with debilitating pain who do not have the financial circumstances to pay for helpful goods and services (e.g., massages, grocery delivery), may struggle more with their arthritis related disabilities. There is a robust association in the literature between low socioeconomic status and severe depressive illness [28]. Poverty, as a result of economic pressures like unemployment and a lack of affordable housing, is more likely to precede serious mental illness like depression and anxiety, operating through lack of opportunity, a reduction in the availability and accessibility of resources, and a higher risk of encountering adversity [29].

White respondents with arthritis in our study were much more likely than racialized respondents to be in a state of both APD and CMH in the bivariate analyses, despite the fact that the whole sample reported that most of their activities were restricted because of pain. These findings are in contrast with Keyes [30] study in the general population that found that while black individuals tend to report lower levels of psychological well-being on measures of life satisfaction and happiness [31], they report higher levels of CMH than white people. The literature is in broad agreement that social contextual factors that reflect exposure to chronic and acute stressors (e.g., racial discrimination) may play an important role in shaping mental flourishing [32]. When we took into account household income and other characteristics, the relationship between racialized status and APD was no longer statistically significant, suggesting disadvantaged financial position, which is more prevalent among racialized individuals, may be somewhat confounding the relationship. However, it is important to note that white respondents still had almost triple the odds of CMH (p < .001) even when household income and other factors were included in the analysis.

It is informative to compare our findings on racial differences to another Canadian study of adults with some pain, attributable to any chronic condition including but not restricted to arthritis. In Gilmour’s [27] study all levels of pain were considered (i.e., mild, moderate, and severe) and individuals who were included whether or not their activities were restricted by pain. In contrast to the large racial disparities in flourishing observed in our study, Gilmour’s [27] study did not find significant racial differences in the odds of flourishing mental health. This suggests that racial differences may be more pronounced among those with the most disabling pain and/or among those with arthritis as opposed to those with other chronic conditions. Further research is needed to provide insight into potential causes of racial discrepancies in flourishing among those with arthritis who are experiencing chronic and disabling pain.

Those with a confidant were more likely to be in both APD and CMH compared to those without a confidant. Confidants are an important source of emotional and instrumental support to help promote and maintain positive mental health outcomes [33]. Older adults who have at least one confidant are less likely to be lonely [34,35]. Furthermore, having a confidant is associated with a wide range of other positive outcomes including fewer physician visits [36], enhanced immunological functioning [37], and reduced stress [38]. Although the exact mechanisms by which a confidant supports mental health are not fully understood, it is thought that a the provision of emotional support can help enhance self-esteem and help the individual buffer general stress [39,40].

Consistent with earlier studies [10], this study found sleep problems to be negatively associated with CMH and APD. This underscores the importance of health professionals asking about sleep problems, particularly as chronic pain can undermine the quality of sleep [41]. Among individuals with chronic pain, cognitive behavioral therapy (CBT) has been shown to significantly reduce insomnia [42]. CBT is an already established effective and relatively rapid treatment for depression and anxiety in the general population [43] and among those with chronic pain [44].

Those without a lifetime history of major depressive disorder and/or generalized anxiety disorder had a substantially higher prevalence of APD and CMH than those with a lifetime history of either disorder. This suggests the need to provide effective treatment. In addition to CBT, other interventions that are promising to deal with mental health issues among individuals experiencing chronic pain, includes operant-behavioral therapy [45], mindfulness-based stress reduction [46], acceptance and commitment therapy [47], and motivational interviewing [48]. In a systematic review and meta-analysis, Knittle, Maes [49] found that self-regulation psychological therapies significantly improved depressive symptoms and anxiety among individuals with rheumatoid arthritis and chronic pain. Technology is also being leveraged to deliver interventions to individuals experiencing chronic pain with encouraging results. Text message-based social support was found to reduce pain perception and interference, and to increase positive affect [50], while web-based mindfulness has been shown to increase life satisfaction [51]. Text- and web-based interventions offer the prospect of improved access to care among individuals experiencing chronic pain.

There are several limitations to this study which should be considered carefully when reviewing the current findings. First, multiple factors that could influence the findings were not controlled for due to these variables not being included in the CCHS-MH. These include the type of arthritis (e.g., osteoarthritis, rheumatoid arthritis), age of onset of arthritis, and how long participants have been coping with arthritis and chronic pain. Any current or past treatments undergone for arthritis or for mental health problems were not available to be analyzed. Future research should include the type, severity, and duration of arthritis while examining their influences on CMH. It would also be beneficial to have chart reviews [52] rather than self-report of a medical diagnosis of arthritis. Another limitation is the cross-sectional design of the study, not allowing for causal inferences to be made. Given the bidirectional relationship between chronic pain and mental illness [53], it would be beneficial for future longitudinal studies to examine the relationship between chronic pain and mental health outcomes among those with arthritis to better understand the direction of the relationship. Finally, the CCHS-MH does not provide information on specific race/ethnicities. As a result, we were unable to examine differences in APD and CMH among different ethnicities, beyond comparing racialized respondents with white respondents. There are substantial differences in experiences across racial/ethnic categories that may be homogenized by a binary race/ethnicity variable. Future research should examine how disparities in mental health outcomes may vary across different groups of racialized respondents.

Despite these limitations, this study provides a strengths-based examination of the mental health of Canadians with arthritis who are in disabling pain using a nationally representative sample. Approximately three quarters of individuals with arthritis who are in chronic and disabling pain, are in APD and more than half of these individuals are in CMH, which demonstrates substantial resiliency in the population. Social support is a particularly important factor associated with better mental health outcomes. Those with sleep problems and a history of psychiatric disorders are at a disadvantage, underlining the importance of providing appropriate treatment for these issues. Of particular concern, impoverished and racialized respondents were much less likely to be in APD or CMH than their more affluent and white peers suggesting the well-documented health inequities observed in the physical health of those with arthritis are also apparent in the mental health of disabled individuals with arthritis. In light of the large observed differences in mental health outcomes, targeted outreach and treatment are warranted for the most vulnerable individuals with arthritis.
